# Acute Cytomegalovirus Pneumonitis in Patient with Lymphomatoid Granulomatosis

**DOI:** 10.3201/eid1704.101035

**Published:** 2011-04

**Authors:** Shang-Gin Wu, Tzu-Hsiu Tsai, Shang-Ju Wu

**Affiliations:** Author affiliations: National Taiwan University Hospital Yun-Lin Branch, Yun-Lin, Taiwan (S.-G. Wu);; National Taiwan University Hospital, Taipei, Taiwan (T.-H. Tsai, S.-J. Wu)

**Keywords:** CMV pneumonitis, viruses, rituximab, lymphomatoid granulomatosis, cryptogenic organizing pneumonitis, letter

**To the Editor:** Lymphomatoid granulomatosis (LYG) involves a B-cell lymphoproliferative process associated with Epstein-Barr virus (*1*). The disease is characterized predominantly by lung involvement, and the pathologic findings show an angiocentric pattern with lymphoid cell clustering. Patient median survival time is ≈14 months (*2*). Treatment commonly consists of corticosteroids and cyclophosphamide, but a literature review did not demonstrate any benefit from corticosteroid therapy, cytotoxic chemotherapy, or radiation (*3*). Rituximab, a monoclonal antibody, targets a B-cell surface molecule, CD20. Recently, several case reports have been published about the effectiveness of rituximab for LYG (*4**,**5*).

We report acute cytomegalovirus pneumonitis in a patient with LYG. In May 2005, a 40-year-old woman with no history of systemic disease had experienced intermittent dry cough for 1 month. Her cough was not associated with any specific environmental exposure or location. Computed tomographic (CT) scan of the chest showed bilateral multiple lung nodules, and a lung biopsy sample showed lymphohistiocytic and lymphoplasmacytic cell infiltrates with some fibrous material. Prednisolone was prescribed for suspected cryptogenic organizing pneumonitis, but her symptoms improved only partially.

One year later, her symptoms worsened. Chest CT scan showed more nodules in both lungs (Figure, panel A). A repeat lung biopsy sample showed large abnormal lymphoid cells clustering in pink-white nodules around and within blood vessel walls. The abnormal lymphocytes were positive for CD20 and Epstein-Barr virus–encoded RNA. The patient was HIV negative, and her bone marrow showed no evidence of lymphoma. Abdominal and pelvic CT scans did not show other abnormal lymph nodes or organ involvement. LYG was diagnosed.

**Figure:**
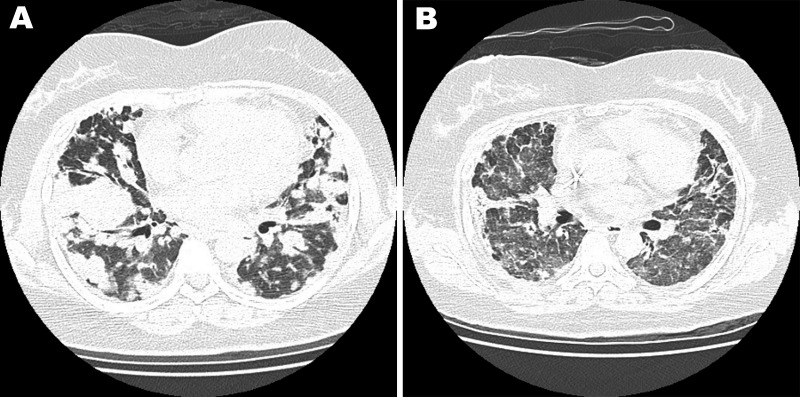
Chest computed tomography scan of a 40-year-old woman with no history of systemic disease. A) Bilateral multiple lung nodules and lymphomatoid granulomatosis were diagnosed after lung biopsy. B) After rituximab treatment, the prior nodular lung lesions decreased dramatically, but newly developed interstitial ground glass opacities appeared.

The patient’s condition did not respond well to induction chemotherapy with cyclophosphamide, doxorubicin, vincristine, and prednisone, and her dyspnea persisted. One week later, rituximab was prescribed; dyspnea dramatically improved after 1 day, and she could tolerate room air without oxygen support. However, 1 day later, severe respiratory distress with hypoxemia suddenly occurred. Although chest CT scan disclosed dramatic resolution of the previously found nodular lung lesions, it also showed newly developed interstitial ground glass opacities (Figure, panel B). A diagnosis of interstitial pneumonitis was considered. Serum cytomegalovirus (CMV) DNA quantification by pp65 gene PCR was performed; the amount was high, up to 2 × 10^5^ copies/mL. Acute CMV pneumonitis was diagnosed. Although the patient received mechanical ventilatory support, CMV intravenous immunoglobulin administration, and ganciclovir therapy, she died 5 days after the onset of acute CMV pneumonitis.

CMV pneumonitis is a common presentation of CMV disease in immunocompromised patients. Host factors, such as presence of cancers or compromised immune function, play a major role in determining pathogenicity of the virus. Respiratory failure is the leading cause of death among patients with rapidly progressive interstitial pneumonitis related to CMV, especially among recipients of renal and bone marrow transplants (*6*). The patient reported here received chemotherapy and steroids; both treatments would render the patient immunocompromised, a condition that may lead to such a rapidly progressive course.

This patient had no identifiable concurrent illnesses that might have been associated with compromised immunity or development of LYG. However, fatal CMV pneumonitis, a complication usually associated with extremely impaired immunity, developed. The case suggests that, even without an identifiable immunocompromised condition, a patient with LYG should be considered an immunocompromised host, and rituximab or other immunosuppressive treatments should be prescribed cautiously; the possibility for rare complications should be recognized.

Because of massive ablation of humoral immunity, the relationship between rituximab and virus infection has been addressed, including varicella–zoster infection, parvovirus B19 infection, and CMV reactivation (*7*). In immunocompromised patients, rituximab might lead to higher risk for virus infection. This issue has been addressed with HIV/AIDS patients with high-grade B-cell lymphoma for whom rituximab is not generally recommended because B-cell ablation could result in more opportunistic infections. For LYG, increased frequency is associated with both congenital and acquired immunodeficiency, such as X-linked lymphoproliferative syndrome, Wiskott–Aldrich syndrome, and HIV/AIDS in which T-cell surveillance is deficient (*8*). Thus, for a patient with LYG whose immune system might be abnormal (*9*), the risks associated with rituximab therapy should be considered the same as the risks for HIV/AIDS patients, and the risk for viral infection or reaction to rituximab should be recognized, particularly in areas where CMV seropositivity in the population is high (*10*). In addition, especially for adult and elderly patients, the gradual increase of CMV seroprevalence with age should be recognized (*10*). Moreover, the patient reported here had previously received cytotoxic drugs as well as maintenance steroid therapy, both of which contributed to a severely compromised immune system. These factors may have led to her acute CMV pneumonitis after receipt of rituximab.

In conclusion, the potential for acute CMV reactivation should recognized during use of rituximab to treat patients with LYG. During rituximab treatment of LYG, routine monitoring for CMV reactivation and other viral infections is warranted.

## References

[1] Liebow AA, Carrington CR, Friedman PJ. Lymphomatoid granulomatosis. Hum Pathol. 1972;3:457–558. 10.1016/S0046-8177(72)80005-4 4638966

[2] Fauci AS, Haynes BF, Costa J, Katz P, Wolff SM. Lymphomatoid granulomatosis. Prospective clinical and therapeutic experience over 10 years. N Engl J Med. 1982;306:68–74. 10.1056/NEJM198201143060203 7053488

[3] Koss MN, Hochholzer L, Langloss JM, Wehunt WD, Lazarus AA, Nichols PW. Lymphomatoid granulomatosis: a clinicopathologic study of 42 patients. Pathology. 1986;18:283–8. 10.3109/00313028609059478 3785978

[4] Jaffre S, Jardin F, Dominique S, Duet E, Hubscher P, Genevois A, Fatal haemoptysis in a case of lymphomatoid granulomatosis treated with rituximab. Eur Respir J. 2006;27:644–6. 10.1183/09031936.06.00086204 16507866

[5] Jordan K, Grothey A, Grothe W, Kegel T, Wolf HH, Schmoll HJ. Successful treatment of mediastinal lymphomatoid granulomatosis with rituximab monotherapy. Eur J Haematol. 2005;74:263–6. 10.1111/j.1600-0609.2004.00367.x 15693798

[6] Meyers JD, Flournoy N, Thomas ED. Risk factors for cytomegalovirus infection after human marrow transplantation. J Infect Dis. 1986;153:478–88. 10.1093/infdis/153.3.478 3005424

[7] Aksoy S, Harputluoglu H, Kilickap S, Dede DS, Dizdar O, Altundag K, Rituximab-related viral infections in lymphoma patients. Leuk Lymphoma. 2007;48:1307–12. 10.1080/10428190701411441 17613758

[8] Jaffe ES, Wilson WH. Lymphomatoid granulomatosis. World Health Organization classification of tumors: pathology and genetics of tumours of hematopoietic and lymphoid tissues. Geneva. Organization. 2001; 185–7.

[9] Wilson WH, Kingma DW, Raffeld M, Wittes RE, Jaffe ES. Association of lymphomatoid granulomatosis with Epstein-Barr viral infection of B lymphocytes and response to interferon-alpha 2b. Blood. 1996;87:4531–7. 8639820

[10] Staras SA, Dollard SC, Radford KW, Flanders WD, Pass RF, Cannon MJ. Seroprevalence of cytomegalovirus infection in the United States, 1988–1994. Clin Infect Dis. 2006;43:1143–51. 10.1086/508173 17029132

